# Supplementary material for: The Evolution and Characterization of the RNA Interference Pathways in Lophotrochozoa
Source: Genome Biol Evol. 2024 May 7;16(5):evae098. doi: 10.1093/gbe/evae098 (PMC11114477; doi:10.1093/gbe/evae098)
Supplement: evae098_Supplementary_Data [file evae098_supplementary_data.zip › Supplementary_materials.zip!/selected_miRNAs/NoGe_selected_miRNAs/known_miRNA_families_pdf_files/NMRB01000055.1_Notospermus_geniculatus_strain_Ushimado_scaffold55,_whole_genome_shotgun_sequence_2176.pdf]

Provisional ID	:	NMRB01000055.1_Notospermus_geniculatus_strain_Ushimado_scaffold55,_whole_genome_shotgun_sequence_2176
Score total	:	2.2
Score for star read(s)	:	-1.3
Score for read counts	:	0
Score for mfe	:	0.5
Score for randfold	:	0
Score for cons. seed	:	3
Total read count	:	1437
Mature read count	:	1257
Loop read count	:	0
Star read count	:	180

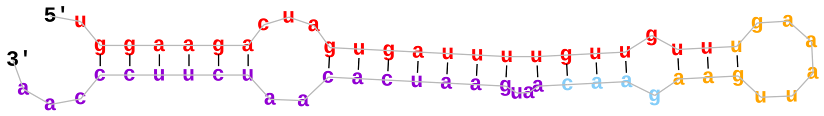

Genome coordinates: NMRB01000055.1 334148-334207

pre-miRNA sequence:
UGGAAGACUAGUGAUUUUGUUGUUUGGAAGACUAGUGAUUUUGUUGUUUGAAAUUGAAGAACAAUGAAUCACAAUCUUCCCAA

Mature sequence: UGGAAGACUAGUGAUUUUGUUGUU

Aligned mature sequence of the *N. geniculatus* pre-miRNA with the top 5 hits from the BLAST search against the pre-miRNA mirGeneDB set:

```
                             ************************
Nge_Mir-7                    uggaagacuagugauuuuguuguu
Lan-Mir-7_5p                 uggaagacuagugauuuuguuguu
Lgi-Mir-7_5p                 uggaagacuagugauuuuguuguu
Npo-Mir-7_5p                 uggaagacuagugauuuuguuguu
Bge-Mir-7_5p                 uggaagacuagugauuuuguuguu
Hme-Mir-7_5p                 uggaagacuagugauuuuguuguu
```
